# Radiogenomic Characterization of PHF2 Expression in Clear Cell Renal Cell Carcinoma: Associations with CT Phenotype and Lipid-Related Imaging Features

**DOI:** 10.3390/cimb48100975

**Published:** 2026-09-23

**Authors:** Federico Greco, Arnaldo Scardapane

**Affiliations:** Department of Experimental Medicine, University of Salento, College ISUFI, via per Monteroni, 73100 Lecce, Italy; arnaldo.scardapane@unisalento.it

**Keywords:** clear cell renal cell carcinoma, kidney cancer, PHF2, radiogenomics, computed tomography, lipid metabolism

## Abstract

Plant homeodomain finger 2 (PHF2) is a histone demethylase involved in epigenetic regulation, tumor suppression, and adipogenic differentiation. Reduced PHF2 expression has been associated with adverse clinicopathological features in clear cell renal cell carcinoma (ccRCC). This study investigated whether PHF2 expression is associated with CT-derived phenotypic characteristics and imaging biomarkers potentially related to lipid metabolism in ccRCC. Clinical, pathological, transcriptomic, and CT imaging data from The Cancer Genome Atlas Kidney Renal Clear Cell Carcinoma (TCGA-KIRC) and The Cancer Imaging Archive (TCIA) were retrospectively matched. Patients were categorized into high- and low-PHF2-expression groups. Morphological CT features, unenhanced tumor attenuation, and abdominal total, visceral, and subcutaneous adipose tissue were assessed. Associations with PHF2 expression and correlations between tumor size and lipid-related imaging parameters were analyzed. A total of 205 patients were included (99 PHF2-high and 106 PHF2-low). Tumors with low PHF2 expression were significantly larger than those with high expression (67.1 ± 32.0 vs. 58.5 ± 30.3 mm, *p* = 0.031). No significant differences were observed in other clinicopathological or CT features, adipose tissue compartments, or tumor attenuation according to PHF2 expression. Tumor size correlated positively with mean (ρ = 0.301, *p* = 0.00007) and minimum (ρ = 0.174, *p* = 0.024) unenhanced CT attenuation. PHF2-low tumors were larger in the unadjusted analysis, although this association was not statistically significant after adjustment for clinicopathological factors. The association between increasing tumor size and higher unenhanced CT attenuation may reflect changes in tumor composition, potentially related to intracellular lipid content, during ccRCC growth.

## 1. Introduction

Radiogenomics investigates the relationship between imaging characteristics and the underlying genomic profile of tumors [1,2]. Imaging manifestations that reflect molecular and biological processes at the tissue level are commonly referred to as radiologic phenotypes [1,2]. Large-scale genomic datasets, including those provided by The Cancer Genome Atlas (TCGA) Research Network, have made it possible to investigate gene expression patterns and genetic alterations across numerous cases of clear cell renal cell carcinoma (ccRCC) [3,4]. Recent advances in transcriptomic and computational approaches have further expanded the ability to characterize complex and heterogeneous gene-expression patterns, providing increasingly detailed molecular information that can potentially be integrated with imaging-derived phenotypes [5]. Radiogenomics provides several important advantages over conventional tissue sampling. Unlike biopsy, it is a non-invasive approach and does not expose patients to procedure-related risks or complications. In addition, while biopsy evaluates only a limited portion of the tumor, radiogenomic analysis can characterize the entire lesion, offering a more comprehensive assessment of intratumoral molecular heterogeneity. This whole-tumor evaluation is particularly valuable for prognostic assessment and treatment planning, since focal biopsy specimens may fail to capture clinically relevant gene expression patterns or genetic alterations that could influence the selection of targeted therapies. Another important strength of radiogenomics is its ability to monitor changes in the molecular profile throughout the course of the disease, enabling longitudinal assessment of genomic alterations in response to treatment. Moreover, imaging-based analyses can simultaneously evaluate multiple lesions within the same patient. Finally, radiogenomics can be integrated with quantitative imaging biomarkers, such as body composition measurements, allowing the investigation of associations between adipose tissue distribution and tumor genomics, as previously demonstrated in patients with clear cell renal cell carcinoma ccRCC [6,7,8,9].

A hallmark of ccRCC is the marked accumulation of intracellular lipids, particularly cholesterol, cholesterol esters, and phospholipids. This metabolic feature is responsible for the tumor’s yellow coloration at macroscopic inspection and for the characteristic clear cytoplasm of neoplastic cells observed on routine hematoxylin–eosin sections [10,11]. ccRCC is increasingly recognized as a malignancy characterized by profound metabolic alterations [12,13]. One of its distinctive features is the prominent accumulation of lipid droplets, although the molecular mechanisms governing their formation and their functional relevance in ccRCC remain incompletely elucidated [14,15,16]. Lipid droplets are dynamic intracellular structures that store lipids and contribute to the regulation of cellular energy homeostasis, thereby playing an important role in the coordination of metabolic pathways [15,17]. Previous investigations focusing on adipose differentiation-related protein (ADFP) have suggested that adipogenic processes and pathways involved in adipocyte differentiation may contribute to ccRCC development and may also be associated with patient prognosis [9,18,19]. Plant homeodomain finger 2 (PHF2) is involved in the epigenetic control of adipogenic differentiation [20,21,22]. This regulatory activity promotes the expression of adipogenesis-associated genes through the removal of methyl groups from dimethylated histone H3 lysine 9 (H3K9me2). Aberrant PHF2 expression or function has been reported across several malignancies, including breast, esophageal, gastric, and colorectal cancers [23,24,25]. PHF2 has been reported to exert tumor-suppressive effects by modulating p53 activity through epigenetic mechanisms, while its expression is decreased in both colorectal and gastric cancer tissues [25].

Previous studies have investigated the role of PHF2 in ccRCC, demonstrating that reduced PHF2 expression is associated with a more aggressive clinicopathological phenotype, including larger tumor size, higher histological grade, more advanced disease stage, and poorer clinical outcomes [26,27]. Moreover, the association between PHF2 expression and the cytoplasmic characteristics of ccRCC has suggested a potential link between PHF2-mediated adipogenic regulation and the distinctive metabolic phenotype of this tumor [27]. However, whether differences in PHF2 expression are reflected in imaging features of ccRCC remains largely unexplored. Based on these findings, we performed a radiogenomic analysis to investigate the relationship between PHF2 expression and computed tomography (CT)-derived phenotypic characteristics in ccRCC. Specifically, we evaluated clinicopathological and morphological CT features, tumor attenuation on unenhanced CT, and abdominal adipose tissue distribution to determine whether PHF2 expression is associated with a distinct imaging phenotype and with imaging biomarkers potentially related to lipid metabolism.

## 2. Materials and Methods

### 2.1. Study Population

This retrospective radiogenomic study was conducted using data from The Cancer Genome Atlas Kidney Renal Clear Cell Carcinoma (TCGA-KIRC) project and The Cancer Imaging Archive (TCIA). Clinical, pathological, radiological, and transcriptomic data were matched on a patient-by-patient basis using the corresponding TCGA identifiers. Patients lacking either preoperative CT imaging or transcriptomic data were excluded from the analysis. Cases with missing information for individual clinicopathological or radiological variables were excluded only from the corresponding statistical analyses. PHF2 gene expression data were obtained from RNA sequencing datasets available through TCGA. PHF2 mRNA expression data were obtained from TCGA via the Genomic Data Commons Data Portal’s Gene Expression Clustering application. These data are log-standardized and centered, with values approximately ranging from −5 to +5, where blue shading reflects expression below the cohort average and red shading reflects expression above the cohort average. To dichotomize PHF2 expression, we used zero (0) as the threshold: samples with expression ≤ 0 were classified as PHF2-low, whereas samples with expression > 0 were classified as PHF2-high. The threshold of 0 was selected because it corresponds to the mean of the standardized expression distribution, thereby distinguishing expression values above the cohort mean from those at or below the cohort mean. This cutoff represents the midpoint of the standardized expression scale and separates lower versus higher PHF2 expressors in a reproducible manner.

### 2.2. CT Image Analysis

All CT images were reviewed and analyzed using Horos software (version 4.0.0 RC2; Horos Project, Annapolis, MD, USA). The CT characteristics assessed for each ccRCC lesion included tumor size, expressed in millimeters, margin appearance (well-defined or ill-defined), and tumor composition (solid or cystic). In solid lesions, the extent of necrosis was also estimated and categorized as 0%, 1–33%, 34–66%, or >66% [6,7,8]. Tumor growth was classified as endophytic, <50% exophytic, or ≥50% exophytic. Additional imaging variables comprised tumor laterality, presence of calcifications, and venous collateral vessels, defined as enlarged renal capsular veins detectable on CT or magnetic resonance imaging [6,7,8]. The assessment also included local tissue infiltration, collecting system involvement, intratumoral hemorrhage, hydronephrosis, renal artery thrombosis, and renal vein thrombosis [6,7,8]. Perirenal fat stranding and thickening of Gerota’s fascia were evaluated separately as binary variables (absent or present) [6,7,8].

Maximum tumor size was obtained by measuring the largest axial diameter on postcontrast CT images [6,7,8]. Lesion margins were classified as well-defined when at least 90% of the tumor circumference could be clearly delineated on postcontrast images, including the interfaces with the renal parenchyma, renal sinus, collecting system, and perinephric fat. Images were evaluated using a window width of 400 HU and a window level of 50 HU [6,7,8].

Tumor composition was determined according to the proportion of cystic tissue. Lesions in which cystic spaces with fluid attenuation ≤20 HU occupied at least 50% of the tumor were categorized as cystic, whereas lesions without cystic areas or with cystic components involving <50% of the tumor volume were classified as solid [6,7,8]. Necrosis was distinguished from cystic components by identifying non-enhancing hypodense areas without a recognizable wall and with poorly defined borders [6,7,8]. In solid tumors, the extent of necrosis was evaluated on nephrographic or excretory phase images [6,7,8].

Calcifications were defined as hyperattenuating plaques or foci. When their nature was uncertain, a maximum attenuation value >60 HU was used to support their classification as calcifications [6,7,8]. Intratumoral hemorrhage was identified by areas within the lesion showing attenuation values compatible with blood, ranging from +30 to +80 HU [6,7,8]. Because attenuation values may overlap between hemorrhage and calcification, equivocal findings were differentiated according to lesion morphology by two radiologists experienced in oncologic imaging (F.G. and A.S., with 13 and 28 years of experience, respectively) [6,7,8].

Infiltration of adjacent tissues was considered present when tumor extension into surrounding normal structures was visible on postcontrast images [6,7,8]. Hydronephrosis was recorded when dilation of the urinary collecting tract was observed on postcontrast examinations [6,7,8]. Renal arterial or venous thrombosis was diagnosed when an intraluminal filling defect was identified within the corresponding vessel on contrast-enhanced images [6,7,8]. Finally, invasion of the collecting system was considered present when a filling defect attributable to tumor involvement was demonstrated during the excretory phase [6,7,8].

### 2.3. Quantitative Assessment of Abdominal Adipose Tissue and Tumor Attenuation

The evaluation of CT-derived features potentially reflecting lipid metabolism in ccRCC included tumor attenuation measurements and quantification of abdominal adipose tissue. Tumor attenuation was assessed on unenhanced CT images by positioning a region of interest (ROI) within the solid component of the lesion with Horos software (version 4.0.0 RC2; Horos Project, USA), and mean, minimum, and maximum Hounsfield Unit (HU) values were recorded. To ensure that the extreme attenuation values were representative rather than isolated measurements, an additional smaller ROI, corresponding to 5% of the original ROI, was used to confirm that these values were present in at least 5% of the sampled tumor area. These measurements were considered indirect indicators of intracellular lipid content. Since adipose tissue generally shows attenuation values between −50 and −100 HU, lower attenuation within the solid tumor component was interpreted as potentially reflecting greater intracellular lipid accumulation [8,9].

Abdominal adipose tissue compartments, including total adipose tissue (TAT), visceral adipose tissue (VAT), and subcutaneous adipose tissue (SAT), were quantified using the semi-automated segmentation tool available in Horos software (version 4.0.0 RC2; Horos Project, USA). The software identifies adipose tissue on CT images according to its characteristic attenuation range, allowing cross-sectional quantification of the different fat compartments. Measurements were expressed as areas (cm^2^) and obtained from a single axial CT image located 3 cm cranial to the inferior endplate of the L3 vertebra, according to the previously described methodology [8,9]. All ROIs and adipose tissue measurements were obtained by consensus between two radiologists (F.G. and A.S., with 13 and 28 years of experience, respectively), who were blinded to the clinical information.

### 2.4. Statistical Analysis

Continuous variables are presented as mean ± standard deviation or median (range), whereas categorical variables are expressed as frequencies and percentages. Comparisons between the PHF2 high-expression and low-expression groups were performed using the Mann–Whitney U test for continuous variables and the chi-square test or Fisher’s exact test for categorical variables, as appropriate. To further investigate the relationship between tumor burden and imaging biomarkers of lipid metabolism, correlations between maximum tumor diameter and abdominal adipose tissue measurements (SAT, VAT, and TAT), as well as tumor attenuation parameters (mean, minimum, and maximum HU values), were evaluated using Spearman’s rank correlation coefficient. Because some clinicopathological and imaging variables contained missing observations, complete-case analysis was performed for each individual variable, resulting in variable-specific sample sizes. As a sensitivity analysis independent of the dichotomization threshold, PHF2 expression Z-score was also analyzed as a continuous variable using Spearman’s rank correlation with maximum tumor diameter and quantitative imaging parameters. To assess whether the association between PHF2 expression and maximum tumor diameter was independent of potential clinicopathological confounders, a multivariable linear regression analysis was performed with maximum tumor diameter as the dependent variable and PHF2 expression status, age, sex, World Health Organization/International Society of Urological Pathology (WHO/ISUP) grade (G1–G2 vs. G3–G4), and American Joint Committee on Cancer T stage (T1–T2 vs. T3–T4) as independent variables. Regression coefficients (β) with 95% confidence intervals (CIs) and *p* values were reported. All statistical analyses were two-sided, and a *p* value < 0.05 was considered statistically significant. Given the number of clinicopathological and imaging variables evaluated and the absence of adjustment for multiple comparisons, these analyses were considered exploratory.

## 3. Results

A total of 205 radiogenomically matched patients with ccRCC were included in the study, comprising 99 patients with high PHF2 expression and 106 patients with low PHF2 expression. Due to incomplete information for some clinicopathological and radiological variables, the number of evaluable patients varied across the analyses.

The baseline clinicopathological and radiological characteristics of the study population are summarized in Table 1. Overall, the two groups were well balanced, and no significant differences were observed in demographic characteristics, pathological findings, tumor morphology, local tumor extension, vascular involvement, abdominal adipose tissue measurements, or unenhanced CT attenuation parameters. A trend toward a higher prevalence of perirenal fat stranding was observed in the low-expression group compared with the high-expression group (59.0% vs. 44.8%, *p* = 0.063), although this difference did not reach statistical significance. In contrast, maximum tumor diameter was significantly greater in tumors with low PHF2 expression than in those with high PHF2 expression (67.1 ± 32.0 mm vs. 58.5 ± 30.3 mm, *p* = 0.031; Figure 1). In a sensitivity analysis treating PHF2 expression as a continuous variable, no significant correlation was observed between PHF2 Z-score and maximum tumor diameter (Spearman’s ρ = −0.086, *p* = 0.225; *n* = 200). Continuous PHF2 expression was also not significantly correlated with the quantitative CT attenuation or adipose tissue parameters (all *p* > 0.05). In multivariable linear regression analysis including 200 patients with complete data, the association between low PHF2 expression and greater maximum tumor diameter was attenuated after adjustment for age, sex, WHO/ISUP grade, and T stage and was no longer statistically significant (β = 5.87 mm, 95% CI −1.91 to 13.64; *p* = 0.138). Conversely, high WHO/ISUP grade (G3–G4) was independently associated with a 10.49-mm greater tumor diameter (95% CI 2.16–18.81; *p* = 0.014), while T3–T4 stage was associated with a 27.13-mm greater tumor diameter (95% CI 18.69–35.57; *p* < 0.001). Age and sex were not significantly associated with maximum tumor diameter.

Clinicopathological and radiological characteristics of patients with clear cell renal cell carcinoma (ccRCC) stratified according to PHF2 expression. Continuous variables are presented as mean ± standard deviation, whereas categorical variables are expressed as number (percentage). Comparisons between the high- and low-expression groups were performed using the Mann–Whitney U test for continuous variables and the chi-square test or Fisher’s exact test for categorical variables, as appropriate. Because some clinicopathological and radiological variables contained missing data, the number of evaluable patients varied among analyses, and percentages were calculated based on the number of available observations for each variable. Abbreviations: WHO, World Health Organization; ISUP, International Society of Urological Pathology; AJCC, American Joint Committee on Cancer.

Quantitative CT-derived imaging biomarkers related to lipid metabolism are summarized in Table 2. Correlation analysis demonstrated no significant associations between maximum tumor diameter and TAT (Spearman’s ρ = −0.018, *p* = 0.804), VAT (Spearman’s ρ = −0.026, *p* = 0.723), or SAT (Spearman’s ρ = 0.018, *p* = 0.810). Conversely, maximum tumor diameter demonstrated a moderate positive correlation with mean unenhanced CT attenuation (Spearman’s ρ = 0.301, *p* = 0.00007; Figure 2A). A weaker but still statistically significant positive correlation was also observed between maximum tumor diameter and minimum unenhanced CT attenuation (Spearman’s ρ = 0.174, *p* = 0.024; Figure 2B). No significant correlation was found between maximum tumor diameter and maximum unenhanced CT attenuation (Spearman’s ρ = 0.114, *p* = 0.140).

Quantitative CT-derived imaging biomarkers related to lipid metabolism according to PHF2 expression and their correlation with maximum tumor diameter in patients with clear cell renal cell carcinoma (ccRCC). Continuous variables are presented as mean ± standard deviation. Comparisons between the high- and low-expression groups were performed using the Mann–Whitney U test. Correlations between maximum tumor diameter and quantitative imaging biomarkers were assessed using Spearman’s rank correlation coefficient. Owing to incomplete quantitative imaging data, the number of evaluable patients varied among variables; therefore, the exact sample size (n) is reported for each analysis. 

## 4. Discussion

The present radiogenomic study investigated the relationship between PHF2 expression and CT-derived phenotypic characteristics in ccRCC, with particular emphasis on imaging biomarkers potentially related to lipid metabolism. The main finding was that tumors with low PHF2 expression showed a significantly larger maximum diameter than those with high PHF2 expression. Conversely, no significant differences according to PHF2 expression were observed for the other clinicopathological and morphological CT features, abdominal adipose tissue compartments, or unenhanced CT attenuation parameters. Interestingly, irrespective of PHF2 expression status, tumor size was positively correlated with mean and minimum attenuation values on unenhanced CT. Taken together, these findings suggest that reduced PHF2 expression may be associated with larger tumor size, while the relationship between PHF2, tumor lipid-related imaging characteristics, and host adipose tissue distribution appears to be more complex.

The association between low PHF2 expression and larger tumor size observed in our study is consistent with previous histopathological evidence. Lee et al. reported that high PHF2 expression was significantly associated with smaller tumor size, lower Fuhrman nuclear grade, lower overall stage, and more favorable cancer-specific and progression-free survival in patients with ccRCC [26]. Similarly, Park et al. found that reduced nuclear expression of PHF2 and C/EBPα was associated with larger tumors, higher WHO/ISUP grade, more advanced pTNM stage, and poorer clinical outcomes [27]. However, in the multivariable analysis, the association between PHF2 expression and tumor diameter was attenuated and no longer statistically significant after adjustment for age, sex, WHO/ISUP grade, and T stage. Furthermore, sensitivity analysis treating PHF2 expression as a continuous variable did not demonstrate a significant correlation with tumor diameter. Taken together, these findings indicate that the observed association between dichotomized PHF2 expression and tumor size should be interpreted cautiously and requires validation in independent cohorts. This finding suggests that the greater tumor size observed in the PHF2-low group may be partly influenced by the more aggressive clinicopathological characteristics of these tumors and should therefore not be interpreted as an independent association with PHF2 expression. Nevertheless, the larger tumor dimensions observed in the PHF2-low group are consistent with the more aggressive clinicopathological phenotype previously associated with reduced PHF2 expression, although this finding requires confirmation in independent cohorts. A possible biological explanation for the association between reduced PHF2 expression and larger tumor size may be related to the tumor-suppressive activity of PHF2. Lee et al. demonstrated that PHF2 interacts with p53 and promotes p53-dependent transcription through epigenetic regulation of chromatin [25]. Specifically, PHF2 facilitates the transcriptional activity of p53 by removing the repressive H3K9me2 histone mark, thereby creating a chromatin environment more permissive to the expression of p53 target genes. Conversely, PHF2 deficiency was associated with impaired activation of downstream p53 signaling, including reduced induction of p21, despite preserved p53 expression [25]. These findings support the hypothesis that reduced PHF2 expression may attenuate p53-mediated tumor-suppressive mechanisms and consequently favor tumor growth. Although these molecular mechanisms were not directly investigated in our cohort, they provide a plausible biological framework for the significantly larger tumor dimensions observed in ccRCCs with low PHF2 expression.

PHF2 is a histone demethylase involved in the epigenetic regulation of adipocyte differentiation, a process in which histone modifications play a critical regulatory role. PHF2 interacts with CCAAT/enhancer-binding protein alpha (CEBPA), a key transcriptional regulator of adipogenesis, and facilitates the expression of CEBPA-responsive adipogenic genes by removing the repressive H3K9me2 histone mark from their promoter regions. Thus, PHF2 functions as a coactivator of CEBPA and contributes to the epigenetic regulation of adipogenesis through its histone demethylase activity [20,21,28]. Despite the established involvement of PHF2 in adipogenic regulation, our study did not identify any statistically significant association between PHF2 expression and CT-derived biomarkers potentially related to lipid metabolism, including tumor attenuation values and abdominal adipose tissue compartments (SAT, VAT, and TAT). These findings suggest that the biological relationship between PHF2 and adipogenesis may not directly translate into measurable differences in tumor attenuation or systemic adipose tissue distribution on CT. Nevertheless, lipid metabolism in ccRCC is regulated by a complex network of molecular pathways, and the absence of a significant association between PHF2 expression and tumor attenuation does not exclude a relationship between the lipid-related molecular profile of ccRCC and its CT phenotype. In this context, proteins involved in fatty acid uptake and lipid droplet formation, such as ADFP, have shown significant associations with CT attenuation in ccRCC. In our previous radiogenomic analysis of low WHO/ISUP grade ccRCC, differences in unenhanced tumor attenuation were observed according to ADFP expression, supporting the potential role of CT attenuation as an imaging biomarker of lipid-related molecular characteristics [9]. This discrepancy may reflect the distinct biological functions of PHF2 and ADFP: PHF2 primarily acts as an upstream epigenetic regulator of adipogenic differentiation, whereas ADFP is more directly involved in intracellular lipid droplet formation and storage. Therefore, the ability of CT attenuation to reflect lipid-related molecular alterations in ccRCC may depend on the specific biological role of the gene or protein investigated rather than representing a uniform imaging manifestation of adipogenic pathway activity.

An additional finding of our study was the positive association between tumor size and unenhanced CT attenuation. In particular, maximum tumor diameter showed a moderate positive correlation with mean attenuation and a weaker correlation with minimum attenuation, whereas no significant relationship was observed with maximum HU values. Considering that lower attenuation values in ccRCC may reflect a greater intracellular lipid component, these findings raise the possibility that tumor lipid composition may vary according to tumor size. The positive correlation between tumor size and unenhanced CT attenuation observed in our cohort may be interpreted in the context of the relationship between tumor progression and intracellular lipid accumulation in ccRCC. Yao et al. showed that ADFP tends to be more highly expressed in low-grade ccRCCs and suggested that its downregulation may accompany tumor dedifferentiation and progression [18]. Consistently, Choi et al. found that low-grade ccRCCs were smaller and showed lower attenuation on unenhanced CT, potentially reflecting their greater intracellular lipid content [29]. A recent radiogenomic study further supported this relationship by demonstrating significantly lower minimum HU values in low-grade ccRCCs expressing ADFP [9]. Taken together, these findings support the hypothesis that the higher tumor attenuation associated with larger tumor size observed in our cohort could reflect changes in tumor composition occurring during ccRCC growth, potentially including a reduction in intracellular lipid accumulation. However, this interpretation remains speculative, as intracellular lipid content was not directly measured in our cohort and CT attenuation may be influenced by other tumor-related and technical factors.

From a radiogenomic perspective, our findings provide further evidence that CT-derived phenotypic characteristics may reflect specific aspects of the molecular biology of ccRCC. In particular, the association between reduced PHF2 expression and larger tumor size suggests that a readily available imaging feature may capture, at least in part, biological differences related to PHF2 expression.

This study has several limitations. First, its retrospective design and the use of data derived from the TCGA-KIRC and TCIA repositories may have introduced selection bias. In addition, CT examinations were acquired across different institutions using heterogeneous imaging protocols, including potential variability in acquisition and reconstruction parameters, which may have influenced quantitative attenuation measurements. Given the retrospective nature of the dataset, retrospective harmonization of these parameters was not feasible. Another limitation is the absence of direct histopathological or biochemical quantification of intracellular tumor lipids. Therefore, although unenhanced CT attenuation may provide an indirect indication of lipid content, its relationship with intracellular lipid accumulation cannot be directly established from our data, and attenuation values may also be influenced by other tumor-related and technical factors. Furthermore, given the number of clinicopathological and imaging variables evaluated and the absence of adjustment for multiple comparisons, the observed associations should be considered exploratory and require validation in independent cohorts. Finally, PHF2 expression was categorized using normalized gene expression values, and the observed associations should be confirmed in independent cohorts. Further studies integrating molecular, histopathological, and quantitative imaging data may provide a more comprehensive understanding of the relationship between PHF2 expression, lipid metabolism, and the imaging phenotype of ccRCC.

## 5. Conclusions

In conclusion, this exploratory radiogenomic study identified an unadjusted association between reduced PHF2 expression and larger tumor size in ccRCC; however, this association was not statistically significant after adjustment for age, sex, WHO/ISUP grade, and T stage. Although PHF2 expression was not directly associated with CT-derived biomarkers potentially related to lipid metabolism, the positive relationship between tumor size and unenhanced CT attenuation raises the hypothesis that changes in tumor composition, potentially including intracellular lipid content, may accompany ccRCC growth; however, this interpretation requires direct histopathological or biochemical validation. These findings highlight the complex interplay between PHF2 expression, tumor growth, and lipid-related imaging characteristics and further support the potential of radiogenomic approaches to improve the characterization of ccRCC biology.

## Figures and Tables

**Figure 1:**
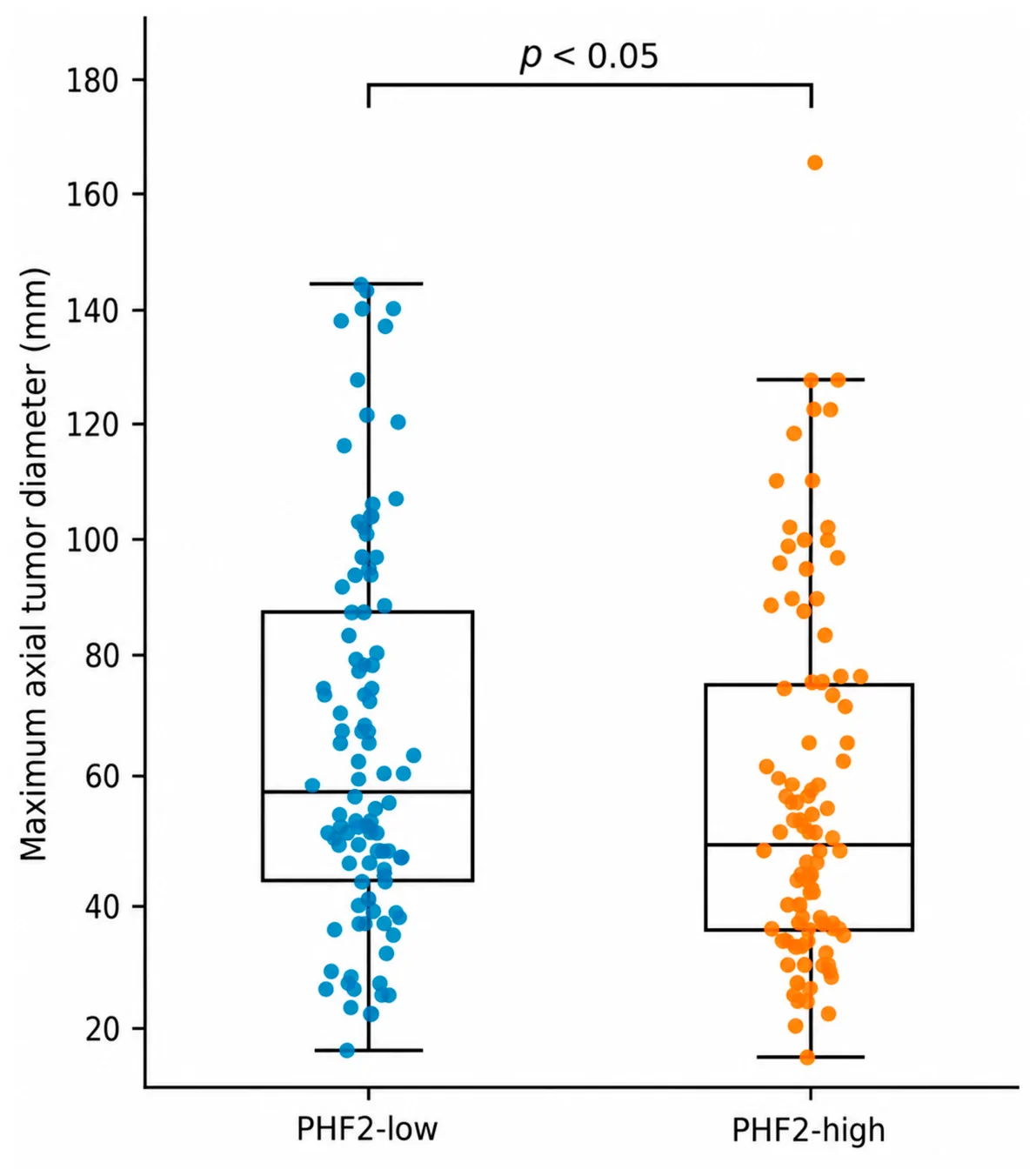
Comparison of maximum tumor diameter between patients with high and low PHF2 expression. Box-and-whisker plots show the distribution of maximum tumor diameter measured on the axial CT plane. The central line represents the median, the box indicates the interquartile range, and the whiskers represent the data distribution. Individual patients are shown as dots. Tumors in the low PHF2 expression group were significantly larger than those in the high PHF2 expression group (*p* < 0.05).

**Figure 2:**
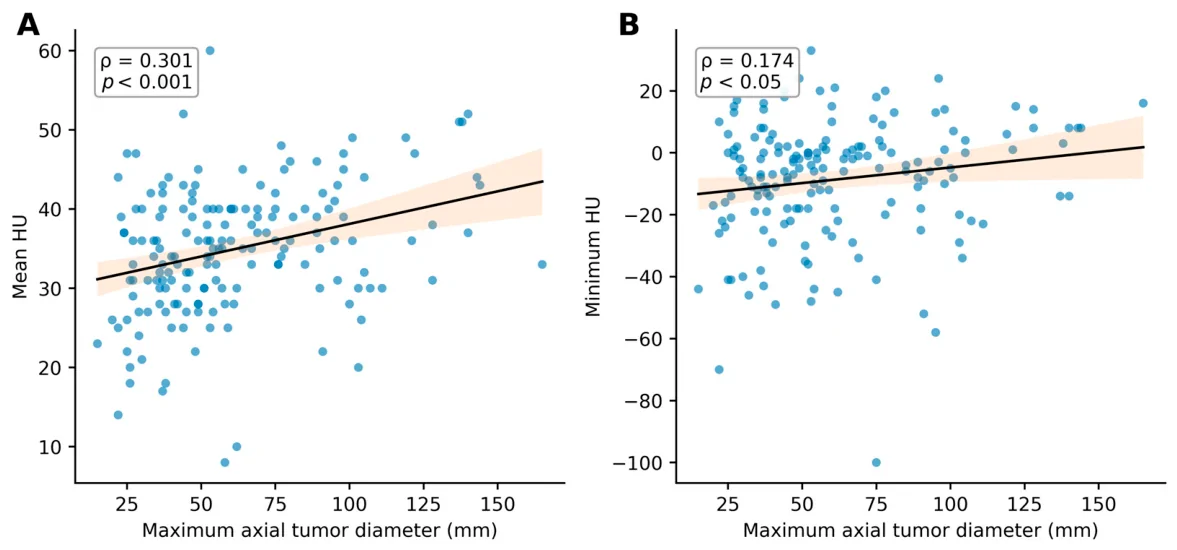
Correlations between maximum tumor diameter and unenhanced CT attenuation in patients with ccRCC. Scatter plots illustrate the relationships between maximum axial tumor diameter and (**A**) mean and (**B**) minimum tumor attenuation measured on unenhanced CT images. Solid lines represent the linear regression fits, and shaded areas indicate the 95% confidence intervals. Maximum tumor diameter showed a moderate positive correlation with mean unenhanced CT attenuation (Spearman’s ρ = 0.301, *p* < 0.001) and a weaker positive correlation with minimum unenhanced CT attenuation (Spearman’s ρ = 0.174, *p* < 0.05).

**Table 1:** Clinicopathological and radiological characteristics of patients with clear cell renal cell carcinoma according to PHF2 expression.

Variable	High PHF2 Expression (*n* = 99)	Low PHF2 Expression (*n* = 106)	*p* Value
**Patients, *n***	99	106	—
**Sex**			0.465
Male	67 (67.7%)	66 (62.3%)	
Female	32 (32.3%)	40 (37.7%)	
Age, years	58.6 ± 12.6	60.9 ± 12.1	0.247
**Ethnicity**			0.439
White	89 (89.9%)	97 (91.5%)	
Black	9 (9.1%)	6 (5.7%)	
Asian	1 (1.0%)	1 (0.9%)	
Other	0 (0.0%)	2 (1.9%)	
**History of other malignancy**			0.329
Yes	22 (22.2%)	18 (17.1%)	
No	77 (77.8%)	87 (82.9%)	
**History of neoadjuvant therapy**			0.390
Yes	8/98 (8.2%)	5/105 (4.8%)	
No	90/98 (91.8%)	100/105 (95.2%)	
**Tumor side**			0.778
Left	44 (44.4%)	49 (47.1%)	
Right	55 (55.6%)	55 (52.9%)	
**WHO/ISUP grade**			0.166
Grade 1	1 (1.0%)	0 (0.0%)	
Grade 2	47 (47.5%)	36 (34.3%)	
Grade 3	39 (39.4%)	51 (48.6%)	
Grade 4	12 (12.1%)	18 (17.1%)	
**AJCC T stage**			0.161
T1	57 (57.6%)	51/103 (49.5%)	
T2	9 (9.1%)	13/103 (12.6%)	
T3	30 (30.3%)	39/103 (37.9%)	
T4	3 (3.0%)	0/103 (0.0%)	
**Maximum tumor diameter on axial plane, mm**	58.5 ± 30.3	67.1 ± 32.0	**0.031**
**Tumor margins**			0.373
Well defined	66/97 (68.0%)	62/101 (61.4%)	
Ill defined	31/97 (32.0%)	39/101 (38.6%)	
**Tumor composition**			1.000
Solid	90/97 (92.8%)	94/101 (93.1%)	
Cystic	7/97 (7.2%)	7/101 (6.9%)	
**Necrosis grade**			0.999
Grade 1	6/97 (6.2%)	6/101 (5.9%)	
Grade 2	57/97 (58.8%)	59/101 (58.4%)	
Grade 3	25/97 (25.8%)	26/101 (25.7%)	
Grade 4	9/97 (9.3%)	10/101 (9.9%)	
**Growth pattern**			0.820
>50% exophytic	64/97 (66.0%)	65/102 (63.7%)	
<50% exophytic	26/97 (26.8%)	31/102 (30.4%)	
Endophytic	7/97 (7.2%)	6/102 (5.9%)	
**Calcifications**			0.372
Presence	16/98 (16.3%)	22/102 (21.6%)	
Absence	82/98 (83.7%)	80/102 (78.4%)	
**Collateral vascular supply**			0.470
Presence	44/94 (46.8%)	41/100 (41.0%)	
Absence	50/94 (53.2%)	59/100 (59.0%)	
**Renal artery thrombosis/infiltration**			0.681
Presence	3/96 (3.1%)	2/98 (2.0%)	
Absence	93/96 (96.9%)	96/98 (98.0%)	
**Renal vein thrombosis/infiltration**			0.782
Presence	7/96 (7.3%)	6/98 (6.1%)	
Absence	89/96 (92.7%)	92/98 (93.9%)	
**Invasion of the collecting system**			0.351
Presence	26/96 (27.1%)	33/98 (33.7%)	
Absence	70/96 (72.9%)	65/98 (66.3%)	
**Intralesional hemorrhage**			0.621
Presence	1/96 (1.0%)	3/98 (3.1%)	
Absence	95/96 (99.0%)	95/98 (96.9%)	
**Hydronephrosis**			1.000
Presence	3/97 (3.1%)	3/99 (3.0%)	
Absence	94/97 (96.9%)	96/99 (97.0%)	
**Perirenal infiltration**			0.679
Presence	3/98 (3.1%)	2/101 (2.0%)	
Absence	95/98 (96.9%)	99/101 (98.0%)	
**Perirenal fat stranding**			0.063
Presence	43/96 (44.8%)	59/100 (59.0%)	
Absence	53/96 (55.2%)	41/100 (41.0%)	
**Thickening of Gerota’s fascia**			0.769
Presence	35/96 (36.5%)	39/100 (39.0%)	
Absence	61/96 (63.5%)	61/100 (61.0%)	

**Table 2:** Quantitative CT-derived lipid metabolism imaging biomarkers according to PHF2 expression and their correlation with maximum tumor diameter.

Variable	High PHF2 Expression	Low PHF2 Expression	*p* Value	Correlation *n*	Spearman’s ρ	Correlation *p* Value
**TAT (cm^2^)**	420.3 ± 199.0 (*n* = 94)	407.3 ± 174.7 (*n* = 97)	0.725	189	−0.018	0.804
**VAT (cm^2^)**	192.5 ± 110.9 (*n* = 94)	206.5 ± 120.4 (*n* = 97)	0.488	189	−0.026	0.723
**SAT (cm^2^)**	227.8 ± 122.5 (*n* = 94)	200.8 ± 96.3 (*n* = 97)	0.175	189	0.018	0.810
**Mean unenhanced CT attenuation (HU)**	34.5 ± 8.4 (*n* = 83)	35.3 ± 8.3 (*n* = 88)	0.256	170	0.301	0.00007
**Minimum unenhanced CT attenuation (HU)**	−6.5 ± 16.6 (*n* = 83)	−10.5 ± 21.3 (*n* = 88)	0.598	170	0.174	0.024
**Maximum unenhanced CT attenuation (HU)**	79.1 ± 17.8 (*n* = 83)	79.7 ± 19.9 (*n* = 88)	0.867	170	0.114	0.140

## Data Availability

The data analyzed in this study are publicly available from TCGA through the Genomic Data Commons Data Portal and from TCIA. No new patient data were generated in this study.
